# *Arabidopsis* SAG protein containing the MDN1 domain participates in seed germination and seedling development by negatively regulating ABI3 and ABI5

**DOI:** 10.1093/jxb/ert343

**Published:** 2013-10-25

**Authors:** Changtian Chen, Changai Wu, Jiaming Miao, Yunxue Lei, Dongxiao Zhao, Dan Sun, Guodong Yang, Jinguang Huang, Chengchao Zheng

**Affiliations:** State Key Laboratory of Crop Biology, College of Life Sciences, Shandong Agricultural University, Taian, Shandong, PR China

**Keywords:** Abscisic acid, AtSAG, drought, MDN1 domain, salt, seed germination

## Abstract

Three proteins containing a midasin homologue 1 (MDN1) domain from the yeast *Solanum chacoense* and *Arabidopsis thaliana* have important functions in yeast survival, seed development, and female gametogenesis. In this study, a novel protein containing the MDN1 domain from *Arabidopsis* negatively regulated abscisic acid (ABA) signalling during seed germination. Seeds of a T-DNA insertion line of this gene exhibited increased *s*ensitivity to *A*BA during seed *g*ermination and seedling development (named *sag*). By contrast, seeds with overexpressed *AtSAG* (OX2) were less sensitive to ABA. The seeds of the *sag* mutant showed similar sensitivity to high concentrations of mannitol and NaCl during these stages. *AtSAG* was also highly expressed in germinating seeds. However, ABA-induced *AtSAG* expression remained almost unchanged. ABA-responsive marker genes, including *ABI3*, *ABI5*, *Em1*, *Em6*, *RD29A*, and *RAB18*, were upregulated in *sag* mutants but were downregulated in OX2. Genetic analyses indicated that the function of AtSAG in ABA signalling depended on ABI3 and ABI5. The expression of some target genes of ABI3 and ABI5, such as seed storage protein and oleosin genes, was induced higher by ABA in *sag* mutants than in wild-type germinated seeds, even higher than in *abi5* mutants. This finding indicated that other regulators similar to ABI3 or ABI5 played a role during these stages. Taken together, these results indicate that AtSAG is an important negative regulator of ABA signalling during seed germination and seedling development.

## Abstract (precis)

*SAG* encodes a MDN1 domain containing protein in *Arabidopsis*. Seeds of a T-DNA insertion line of this gene exhibited hypersensitivity to ABA, mannitol and NaCl during seed germination and early seedling development. SAG was genetically epistatic to ABI3 and ABI5

## Introduction

Seed germination marks the beginning of a new growth cycle in higher plants and is subject to complex mechanisms of regulation by both internal and environmental signals (Bewley, 1997; Finch-Savage and Leubner-Metzger, 2006). For instance, abscisic acid (ABA) is a phytohormone that functions in plant seed germination, seedling growth, and stress tolerance (Koornneef *et al.*, 1989; Leung and Giraudat, 1998; Finkelstein *et al.*, 2002). Molecular genetics approaches have revealed several proteins that function in ABA signalling during seed germination (Finch-Savage and Leubner-Metzger, 2006; Holdsworth *et al.*, 2008; Penfield and King, 2009). For example, ABA-insensitive1 (ABI1) (Leung *et al.*, 1994) and ABI2 (Rodriguez *et al.*, 1998) are protein phosphatases that negatively regulate ABA signalling during seed dormancy and germination. By contrast, ABI transcription factors, such as ABI3 and ABI5, can positively regulate ABA signalling during seed development and germination (Finkelstein *et al.*, 2002). In particular, ABI5 is a basic leucine zipper (bZIP) transcription factor (Finkelstein and Lynch, 2000) that elicits enhanced response to exogenous ABA during germination, seedling development, and subsequent vegetative growth (Lopez-Molina *et al.*, 2001; Finkelstein *et al.*, 2002). ABI5 physically interacts with ABI3 (Nakamura *et al.*, 2001) and is genetically epistatic to ABI3 (Lopez-Molina *et al.*, 2002; Nakashima *et al.*, 2009). ABI5 is required in germination and post-germination growth arrest checkpoint (Lopez-Molina *et al.*, 2001). Some regulators that control ABA sensitivity are mediated by ABI5. For instance, three *Arabidopsis* SnRK2 protein kinases, namely SRK2D, SRK2E, and SRK2I, are involved in ABA signalling through ABI5 phosphorylation (Nakashima *et al.*, 2009). Overexpression of two coupled components of the mitogen-activated protein kinase cascade, MdMPK1 and MdMKK1. from apple leads to ABA sensitivity by ABI5 phosphorylation during transgenic *Arabidopsis* seed germination (Wang *et al.*, 2010).

The midasin homologue 1 (MDN1) domain contains a von Willebrand A (VWA) domain and an *a*denosine triphosphatases *a*ssociated with diverse cellular *a*ctivities (AAA) domain. Proteins containing those domains have important functions during development. For instance, the yeast MDN1 domain-containing protein Rea1 functions in ribosome maturation (Talkish and Woolford, 2009; Bassler *et al.*, 2010). ScMDN1, a plant homologue of Rea1, is involved in seed and shoot development in *Solanum chacoense* (Chantha and Matton, 2007). AtMDN1 from *Arabidopsis* is essential for female gametophyte development (Chantha *et al.*, 2010). Furthermore, VWA and AAA domain-containing proteins mediate protein–protein interactions involved in the assembly of complexes, such as ribosomes, proteasomes, and chloroplasts (Snider *et al.*, 2008). For example, *Arabidopsis* Rpn10 contains the VWA domain at the N-terminal and functions as a component of 26S proteasome by recognition of multiubiquitin of specific proteins, such as ABI5 (Voges *et al.*, 1999). The chloroplast Mg chelatase subunit D, which contains one AAA domain and one VWA domain (Whittaker and Hynes, 2002), plays a role in plant responses to ABA (Du *et al.*, 2012). However, MDN1 domain-containing proteins related to ABI3 and ABI5 have not yet been reported.

The current study reports another MDN1 domain-containing AtSAG protein from *Arabidopsis* that negatively regulated ABA sensitivity during seed germination and seedling development. The possible regulatory mechanisms are discussed according to present data, bioinformatics, and related literature.

## Materials and methods

### Plant materials and growth conditions

Seeds of each genotype *Arabidopsis thaliana* from Columbia (Col-0) background were harvested at the same time from plants grown under the same conditions. Seeds were surface sterilized with 70% ethanol for 5min, incubated in 2.6% sodium hypochlorite for 10min, and washed five times with sterile water. Germination assays were carried out with three replicates of 100 seeds. The sterile seeds were plated on half-strength Murashige and Skoog (MS) medium containing 1% (w/v) sucrose and 0.8% agar (control). The seed-dotted plates were maintained in the dark at 4 °C for 3 d to break dormancy (stratification) and then transferred to a growth chamber under 16/8 light/dark conditions at 22 °C.

### Verification of the single and double mutants

The *sag* mutant (Salk_013481), containing a T-DNA insertion in the exon of *AtSAG*, was bought from *Arabidopsis* Biological Resource Center (ABRC). The *abi5* mutant (Salk_013163C) was a gift from Dr Yinggao Liu (Shandong Agricultural University, China). To determine whether the mutant line was homozygous, PCR was performed using genomic DNA with the following gene-specific primers: for *sag*, *abi5* LP and RP and one specific primer LBb1.3 (Supplementary Table S1, available at *JXB* online).

The *sag/abi5* double mutant was constructed by crossing the two single mutants. The double mutant was identified by PCR based on the genotype of the *AtSAG* locus and the confirmed sequence of the *ABI5* locus.

### Construction and generation of transgenic plants

To construct 35S:AtSAG, the *AtSAG* coding sequence was amplified using Col-0 cDNA by PCR with gene-specific primers (Supplementary Table S1). The resulting PCR product was cloned into the *Sal*I and *Kpn*I sites of binary vector pBI121 under the control of the cauliflower mosaic virus 35S promoter.

To generate a AtSAG-RNAi construct, *Arabidopsis* pFGC5941 vector (ABRC) for dsRNA production was used. A 360-bp fragment of *AtSAG* cDNA was amplified by PCR using gene-specific primers RNAi-F and RNAi-R (Supplementary Table S1). The fragment was initially cloned between *Asc*I and *Swa*I sites before an inverted repeat of the same fragment was inserted between *Bam*HI and *Xba*I sites of pFGC5941.

A 1550-bp promoter sequence was amplified from genomic DNA by PCR and verified by sequencing to construct pSAG:GUS. The PCR fragment was cloned into the *Hin*dIII and *Bam*HI sites of PBI121 to obtain the construct containing the *AtSAG* native promoter fused in the β-glucuronidase (GUS) coding region. The primers used were GUS-F and GUS-R (Supplementary Table S1).

The transformation of *Arabidopsis* plants was performed by a floral dip infiltration method using *Agrobacterium tumefaciens* GV3101 (EHA105). T_2_ seeds from each selected transgenic plant were plated on half-strength MS medium containing 50mg l^–1^ kanamycin (for PBI121) or 10mg l^–1^ phosphinothricin (for pFGC5941) as selective antibiotics to select the homozygous lines.

### Histochemical GUS staining

Histochemical localization of GUS activities in the transgenic seedlings or germinated seeds were analysed after the transgenic plants had been incubated overnight at 37 °C in 1mg ml^–1^ 5-bromo-4-chloro-3-indolyl-glucuronic acid, 5mM potassium ferrocyanide, 0.03% Triton X-100 and 0.1M sodium phosphate buffer, pH 7.0. Then the tissues were cleaned with 70% ethanol. The cleaned tissues were then observed and pictures were taken by stereoscope. To examine the detailed GUS staining, the tissues were observed with a bright-field microscope and photographed. The GUS staining data were representative of at least five independent transgenic lines for each construct.

### Seed germination and cotyledon greening

Plants of different genotypes were grown in the same conditions, and seeds were collected at the same time. For each comparison, seeds were planted on the same plate containing half-strength MS medium without or with different concentrations of ABA, 200mM NaCl, and 500mM mannitol. Plates were chilled at 4 °C in the dark for 3 d (stratified) and moved to 16/8 light/dark conditions at 22 °C. Seed germination and cotyledon greening were scored at the indicated times. Germination was defined as an obvious emergence of the radicle through the seed coat. Cotyledon greening is defined as obvious cotyledon expansion and turning green.

### RNA extraction

For RNA isolation, the plant tissues grown after 3-d stratification for the indicated times in a growth chamber under 16/8 light/dark conditions at 22 °C were separately harvested, frozen in liquid nitrogen, and stored at –80 °C until use. Total RNA was isolated from different *A. thaliana* seedlings using a universal plant total RNA extraction kit (spin-column)-I (BioTeke, Beijing, China).

### Real-time PCR analysis

cDNA was synthesized using PrimeScript RT (reverse transcriptase) with oligo-dT primer using the PrimeScript RT master mix kit (Takara). All samples were prepared to a final volume of 10 µl. A SYBR green real-time PCR master mix (Takara) and a Chromo 4 real-time PCR detector (Bio-Rad) were used. The primers used to amplify *AtSAG* and the other genes were designed based on sequences downloaded from the TAIR database (http://www.arabidopsis.org/). Real-time PCR experiments were performed at least thrice under similar conditions with EF1-α as an internal control. The primers are shown in Supplementary Table S1. Although EF1-α was reported as ABA-inducible in the micropylar endosperm or/and radicle (Graeber *et al.*, 2011), the data showed that, during seed germination, it was induced less than 1.5-times in 1-d-old germinating seedlings, and its expression pattern was similar in the presence or absence of ABA (data not shown). Furthermore, when actin2 was used as the internal control for qRT-PCR, a similar pattern of gene expression was obtained between the wild type (WT) and the *sag* mutant with or without ABA.

## Results

### Isolation of the *sag* mutant

**Fig. 1.**
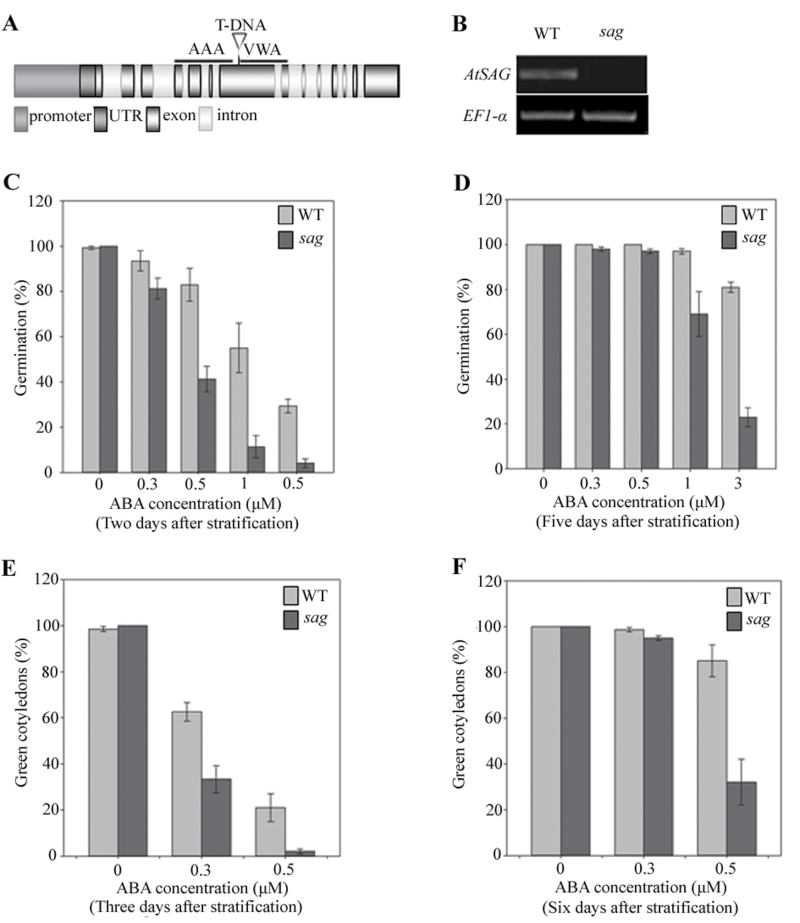
Characterization and ABA-responsive analysis of the T-DNA insertion mutant of *sag* plants. (A) T-DNA insertion site in *sag*; black boxes represent exons; white boxes represent introns; AAA and VWA represent the AAA and VWA domains in the putative peptide. (B) Reverse-transcription PCR analysis to confirm the knockout status of *sag*; upper panel shows *AtSAG* expression (35 cycles) in wild type (WT) and mutant line; lower panel shows *EF1-α* expression (25 cycles) as a control. (C and D) Seed germination records of WT and *sag* mutants treated with 0, 0.3, 0.5, 1.0, and 3.0 μM abscisic acid (ABA) at 2 and 5 d after stratification, respectively. (E and F) Cotyledon greening rates of the germinated seeds described in C and D with 0, 0.3, and 0.5 μM ABA at 3 and 6 d after stratification, respectively. Data are mean ± SD of at least three replicates; at least 100 seeds per genotype were counted in each replicate.

To find other novel regulators and to expand ABA signalling networks during seed germination and abiotic stresses, various T-DNA insertion mutants purchased from the ABRC were screened on half-strength MS medium containing 0.5 µM ABA during seed germination. The mutant sensitive to ABA during seed germination is called *sag*. A *sag* mutant was produced by knocking out a MDN1 domain-containing protein (Fig. 1A, B, Supplementary Fig. S1). T-DNA was inserted after nucleotide 938 (Fig. 1A). This procedure may result in VWA domain deletion (Supplementary Fig. S1: asterisks for VWA domain and a arrowhead for T-DNA insertion site).

### *sag* seeds are hypersensitive to ABA during seed germination and seedling establishment

In the absence of ABA, no obvious differences were observed in germination rates between WT and *sag* seeds (Fig. 1C, D). At 0.3 and 0.5 µM ABA, while the germination rates of WT seeds were 94 and 83% at 2 d of germination, respectively, the germination rates of *sag* seeds were 81 and 41%, respectively (Fig. 1C). At 1.0 and 3.0 µM ABA, while the germination rates of WT seeds were 97 and 81% at 5 d of germination, respectively, the germination rates of *sag* seeds were 69 and 23%, respectively (Fig. 1D).

The early seedling growth of *sag* mutants was also slower than that of WT (Fig. 1E, F). A maximum of 100% green cotyledons were observed in both WT and *sag* seedlings after 3 d of germination in the absence of ABA. At 0.3 µM ABA, 62% of WT but only 33% of *sag* mutants had green cotyledons after 3 d of germination. At 0.5 µM ABA, 21% of WT but only a few *sag* seedlings had green cotyledons after 3 d of germination. After 6 d of germination, 32% of *sag* seedlings and 85% of WT seedlings had green cotyledons. These results indicated that *sag* mutants were hypersensitive to ABA during seed germination and seedling development.

### Higher expression levels of *AtSAG* during germination were not induced by ABA in *Arabidopsis*

**Fig. 2.**
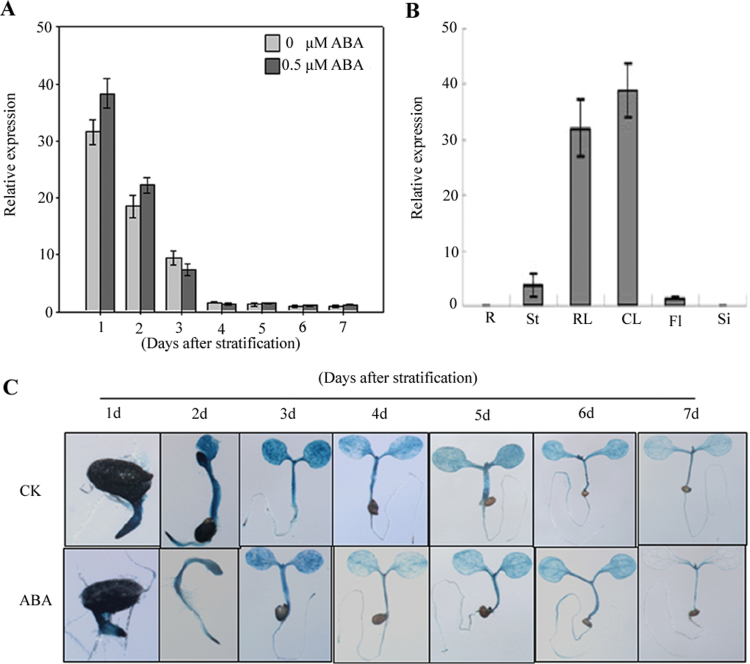
Expression pattern of *AtSAG*. (A) Relative expression of *AtSAG* during and after germination after the end of stratification in WT plants treated with 0 or 0.5 μM ABA. (B) Relative expression of *AtSAG* at differential tissues from the same growth stage in the WT plants. R, root; St, stem; RL, rosette leaf; CL, cauline leaf; F, flower; Si, silique. (C) GUS staining of the pAtASG::GUS transgenic germinating and germinated seedlings grown for 1–7 d on half-strength MS medium containing 1 or 0.5 μM ABA (this figure is available in colour at *JXB* online).

To determine the expression pattern of *AtSAG* during germination in *Arabidopsis*, real-time RT-PCR was carried out. Fig. 2A shows that the expression levels of *AtSAG* were very high at 1–3 d of germination. As germination continued, the expression levels of *AtSAG* decreased. After 4 d of germination, the expression levels of *AtSAG* became much lower. The expression levels of *AtSAG* were not evidently changed by ABA. To further analyse the *AtSAG* expression pattern, GUS activity driven by native promoter of *AtSAG* was detected in pSAG:GUS transgenic plants. Strong GUS staining was observed in seeds germinated at 1, 2, and 3 d, but weak staining was observed in 4- to 7-d-old seedlings (Fig. 2C). ABA treatment did not change the GUS staining pattern during the investigated time points (Fig. 2C), although a ABA-responsive element (ABRE-like) was observed in the promoter region of *AtSAG* (data not shown). Real-time RT-PCR analysis revealed *AtSAG* expression in multiple organs of more mature plants (Fig. 2B). These results suggested that *AtSAG* was not induced by ABA during seed germination and may function in other developmental stages under specific conditions.

### Response of *sag* mutant to ABA defined a limited developmental period

**Fig. 3.**
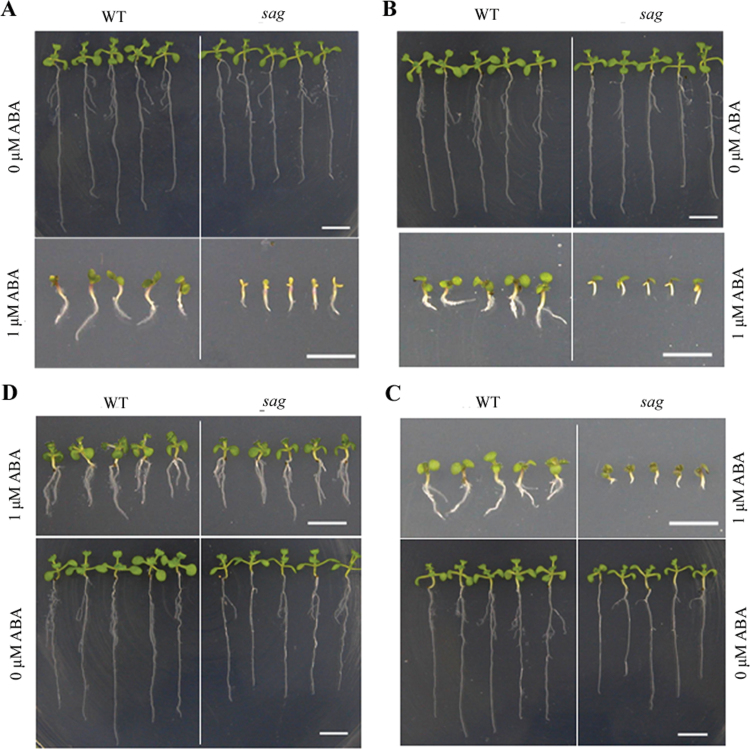
Response of *sag* plants to abscisic acid (ABA) defines a limited developmental period. (A) Wild-type (WT) and *sag* seedlings stratified and germinated on medium containing 0 or 1.0 μM ABA. (B) WT and *sag* seedlings transferred immediately to medium containing 0 or 1.0 μM ABA after stratification on control medium. (C) WT and *sag* seedlings transferred to the medium containing 0 or 1.0 μM ABA at 1 d of germination after stratification on control medium. (D) WT and *sag* seedlings that were transferred to the medium containing 0 or 1.0 μM ABA at 2 d of germination after stratification on control medium (this figure is available in colour at *JXB* online).

Considering the ABA sensitivity of *sag* mutant seeds, the effect of ABA on seed germination and seedling development was investigated. When *sag* seeds were transferred to ABA-containing medium immediately after stratification without ABA, they also showed sensitivity to ABA compared with WT seeds (Fig. 3B). By contrast, the *sag* seeds showed less sensitivity than those directly stratified and germinated seeds on ABA-containing medium (Fig. 3A). When *sag* seeds were transferred to ABA-containing medium after 1 d of germination without ABA, they also showed sensitivity to ABA (Fig. 3C). However, when seeds were transferred to ABA-containing medium after 2 d of germination without ABA, no obvious difference was observed between WT and *sag* seedlings (Fig. 3D). The mutant displayed similar phenotypes in terms of morphology, growth, or development (data not shown). Therefore, AtSAG was involved in ABA responses in seed germination and early seedling development.

**Fig. 4.**
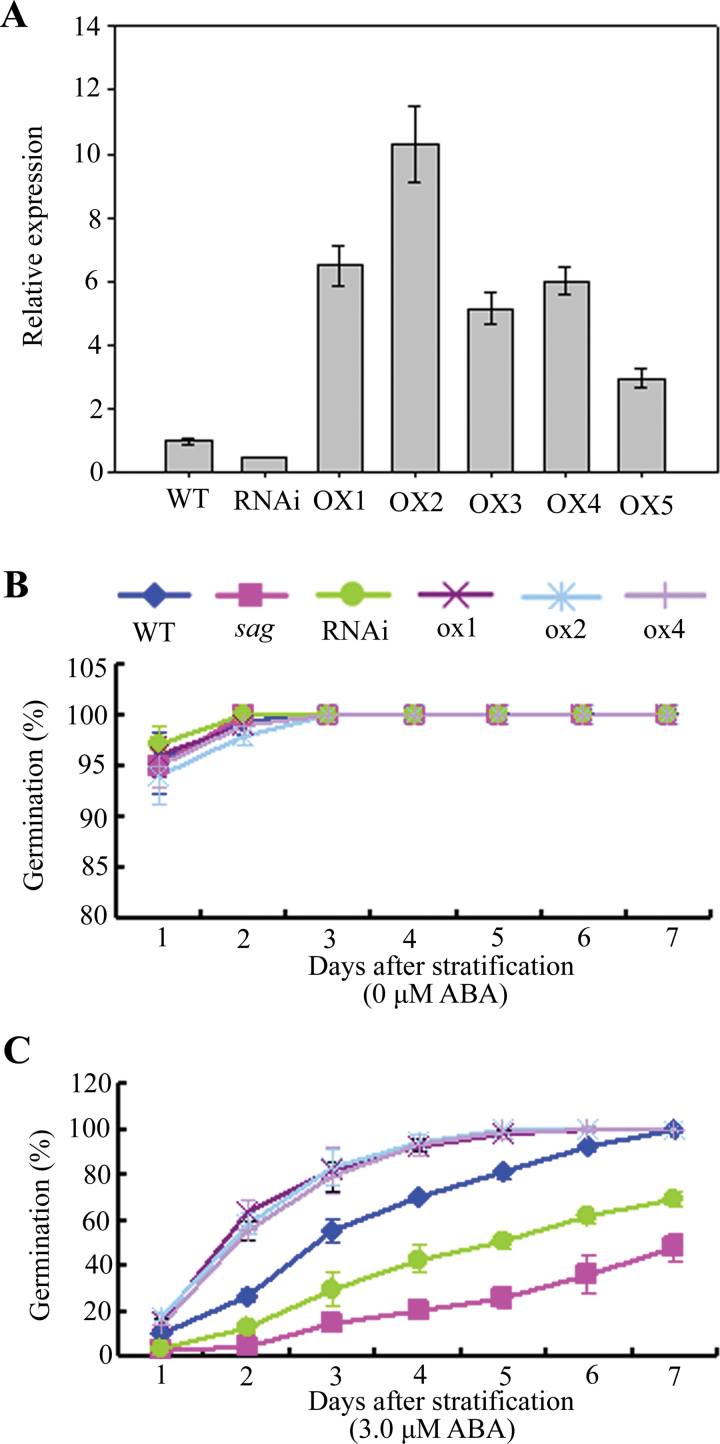
Expression analysis and abscisic acid (ABA) responses of the RNAi and OX lines of *AtSAG*. (A) Real-time PCR analysis of one RNAi line and five independent OX lines of *AtSAG*; cDNA was obtained from total RNA of 10-d-old seedlings of each phenotype; gene expression was normalized to the WT expression level, which was assigned as a value of 1; standard errors are shown as bars above the columns. (B and C) Seed germination of WT, RNAi, and three OX lines during the time course with 0 and 3.0 μM ABA respectively. Data are mean ± SD of at least three replicates; at least 100 seeds per genotype were counted in each replicate (this figure is available in colour at *JXB* online).

To confirm the function of AtSAG in ABA responses, AtSAG-RNAi transgenic plants (RNAi) and AtSAG-overexpressing plants (OX) were generated. AtSAG expression in these lines was assessed by real-time RT-PCR (Fig. 4A). The AtSAG-OX lines showed decreased sensitivity to ABA, whereas the RNAi line showed less sensitivity to ABA than *sag* mutant (Fig. 4C), demonstrating that AtSAG functioned as a negative regulator in ABA response during seed germination and early seedling development.

### AtSAG functioned upstream of ABI5 during germination and seedling development

**Fig. 5.**
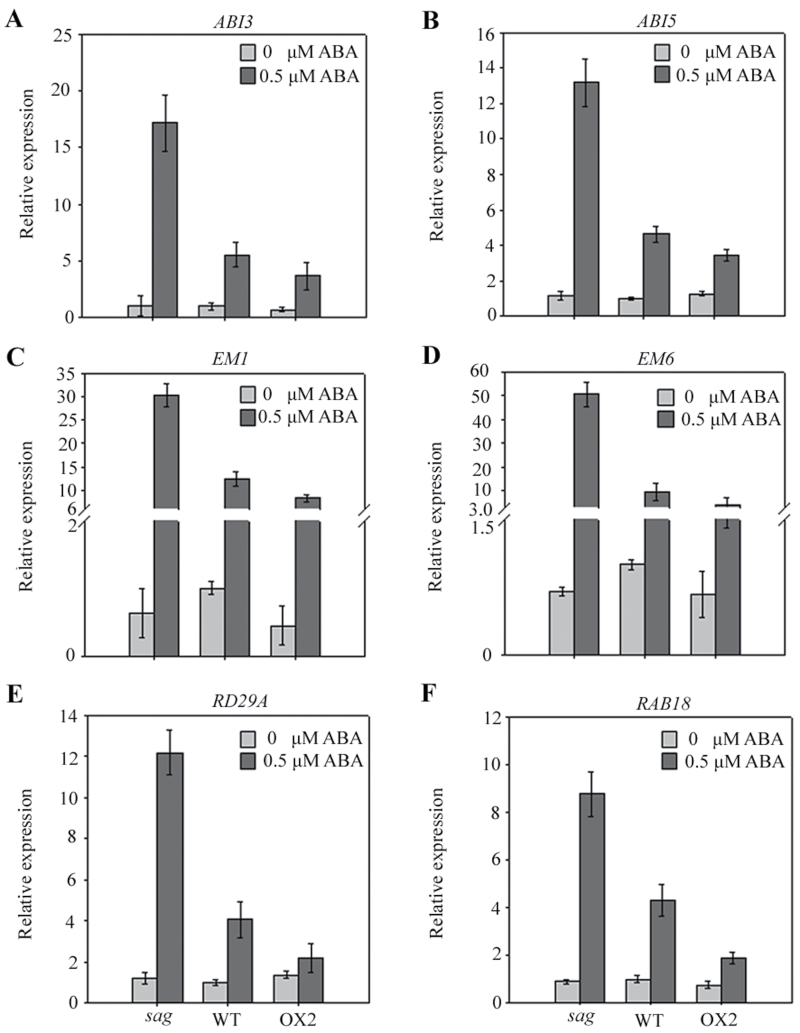
Expression analysis of downstream genes in abscisic acid (ABA) signalling: relative expression of *ABI3* (A), *ABI5* (B), *Em1* (C), *Em6* (D), *RD29A* (E), and *RAB18* (F) in the *sag*, WT, and OX2 seeds germinated for 2.5 d on medium containing 0 or 0.5 μM ABA.

ABI3, ABI5, and late embryogenesis genes were reactivated by ABA within a short development period. To determine whether ABI3 and ABI5 mediated this process, the expression of these genes and their target genes were detected in WT, OX2, and *sag* seeds germinated for 2 d with or without 0.5 µM ABA by real-time RT-PCR. Fig. 5A–F shows that the expression of *ABI3*, *ABI5*, *Em1*, *Em6*, *RD29A*, and *RAB18* were very low and showed no obvious differences among WT, OX2, and *sag* mutants after the seeds were germinated for 2 d without ABA. By contrast, the expression levels of the detected genes, in the presence of 0.5 µM ABA, remarkably increased in *sag* mutants but decreased in OX2 lines. Western blot analysis also showed that ABI3 and ABI5 proteins accumulated at a higher extent in the presence of 0.5 µM ABA in *sag* mutant seeds than in WT seeds (Supplementary Fig. S2). These results suggested that AtSAG may function upstream of ABI3 and ABI5 in ABA signalling during seed germination and seedling development.

**Fig. 6.**
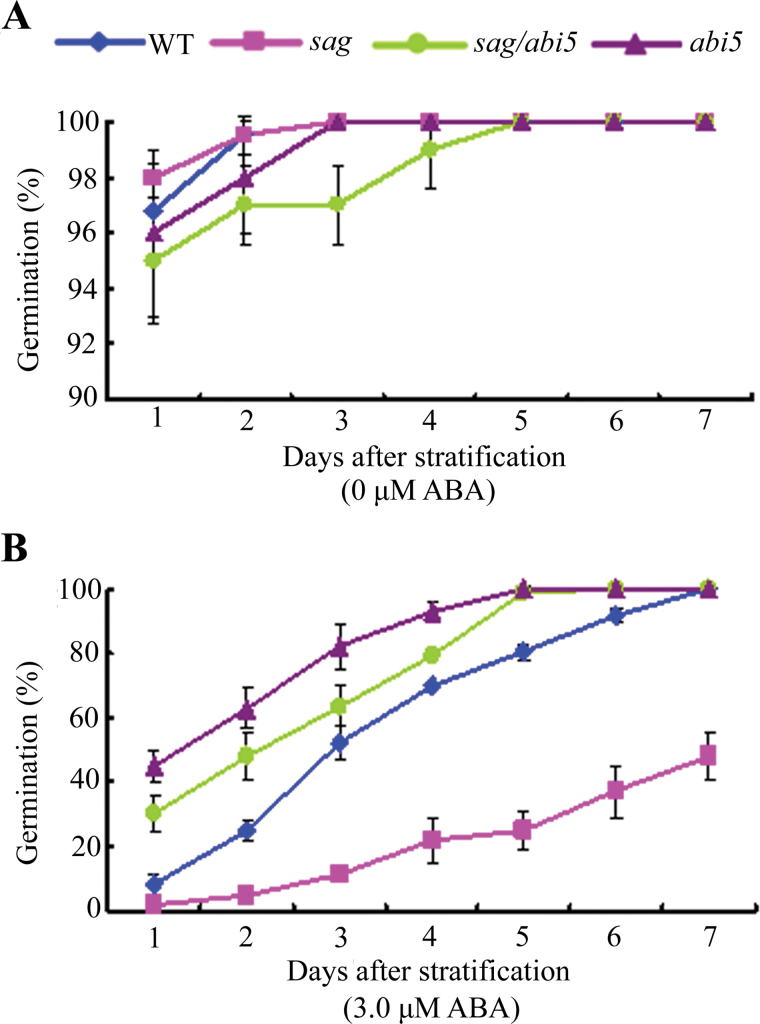
Abscisic acid (ABA) responses of WT, *sag*, *sag/abi5*, and *abi5* germinated seeds: germination rates on half-strength MS medium containing 0 (A) or 3.0 (B) μM ABA during the time course, respectively. Data show the mean ± SD of at least three replicates; at least 100 seeds per genotype were counted in each replicate (this figure is available in colour at *JXB* online).

To determine whether AtSAG functioned upstream of ABI5, the *sag/abi5* double mutant was generated. In general, the double mutant responded similarly to ABA to *abi5* as if AtSAG functioned upstream of ABI5. As expected, the ABA response assays (Fig. 6B) indicated that the *sag/abi5* double mutant was more insensitive to ABA than the *sag* mutant, but exhibited similar sensitivity to *abi5* in the presence of 3 µM ABA. These results suggested that AtSAG functioned upstream of ABI5 in ABA signalling.

### AtSAG participated in the regulation of seed storage protein and oleosin genes during germination and seedling development

**Fig. 7.**
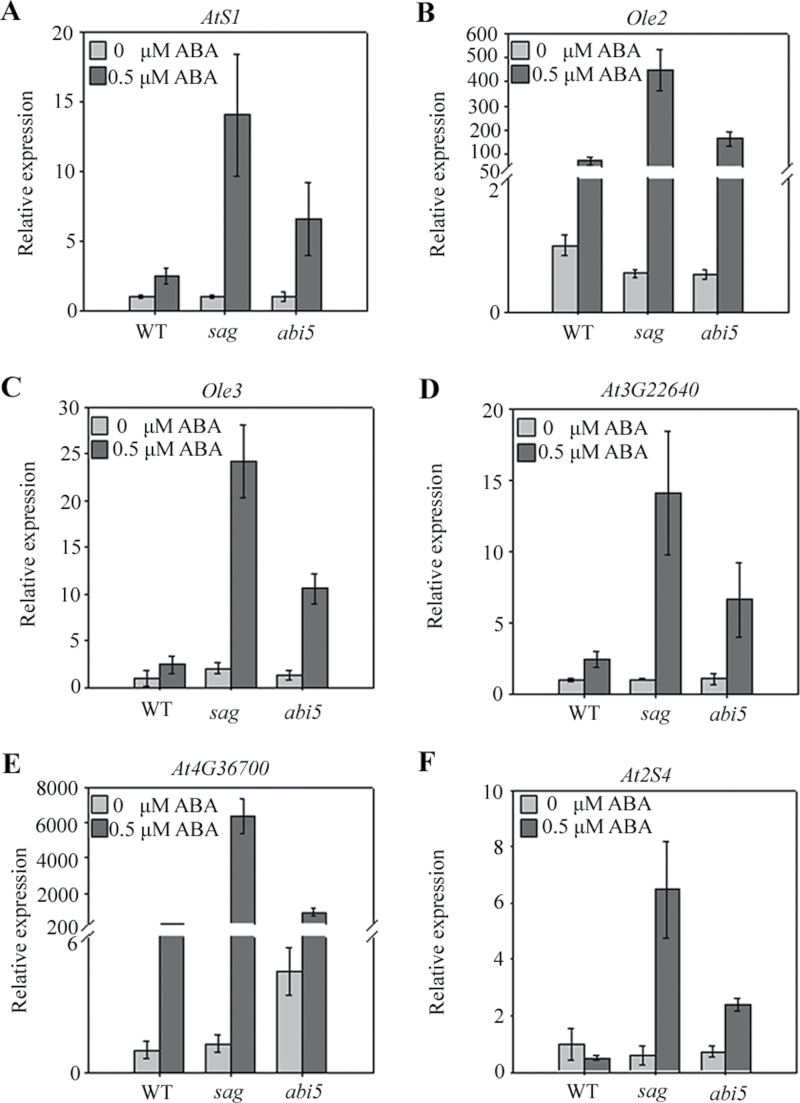
Expression analysis of genes encoding for oleosin and seed storage proteins: relative expression of *AtS1* (A), *Ole2* (B), *Ole3* (C), *At3G22640* (D), *At4G36700* (E), and *At2S4* (F) in WT, *sag*, and *abi5* seeds germinated for 2.5 d on half-strength MS medium with 0 or 0.5 μM abscisic acid (ABA).

The expression of genes encoding seed storage proteins and oleosins are induced by ABI3 during seed development (Crowe *et al.*, 2000; Lara *et al.*, 2003). They may be induced in *sag* mutants. To illustrate this phenomenon, three genes encoding for oleosins and three genes encoding for seed storage proteins were selected for real-time RT-PCR. The expression of these selected genes showed no evident change in *sag* and WT plants without ABA treatment, but their expression was induced more in the *sag* mutants than in the WT after ABA treatment (Fig. 7). Unlike the induced expression of these genes by ABI3, the expression of these genes was induced in *abi5* mutants. Expression of the selected genes was higher in the *sag* mutant than in the *abi5* mutant (Fig. 7). These results demonstrated that the selected genes can be suppressed by AtSAG partially through ABI5.

### Salt and osmotic responses of *sag* and OX2 plants during seed germination and seedling development

**Fig. 8.**
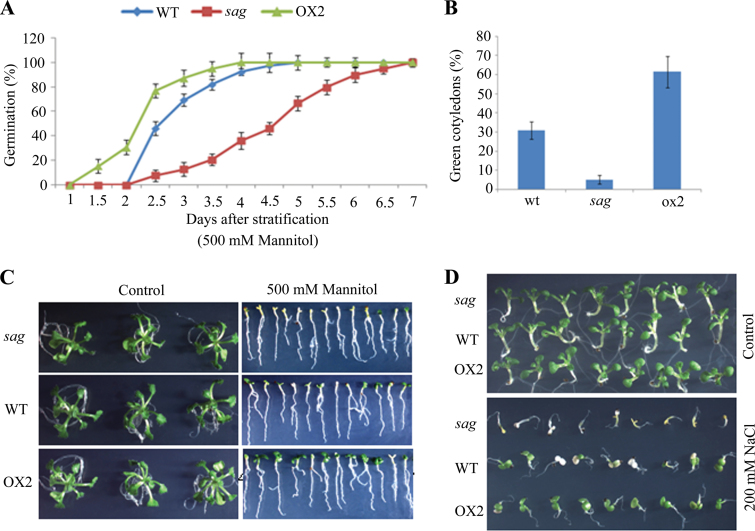
Osmotic and salt responses of the *sag* and OX2 plants during seed germination. (A and B) Germination and cotyledon greening rates of the WT, *sag*, and OX2 plants with 500mM mannitol during the time course. Data show the mean ± SD of at least three replicates; at least 100 seeds per genotype were counted in each replicate. (C and D) WT, *sag* and OX2 seedlings grown on half-strength MS medium containing 500mM mannitol or 200mM NaCl, respectively (this figure is available in colour at *JXB* online).

Plants respond to abiotic stresses, such as drought, salt, and dehydration, and such responses are mediated by ABA signalling. To determine whether AtSAG was regulated in response to abiotic stresses, seeds of *sag*, OX2, and WT plants were sown on a medium containing 500mM mannitol or 200mM NaCl. Germination and green cotyledon rates were scored. The germination and green cotyledon rates of the OX2 lines were higher than those of WT seeds in medium supplemented with 500mM mannitol and the germination and green cotyledon rates of the *sag* mutants were much lower than those of WT seeds (Fig. 8A–C). The seed germination and green cotyledon rates of the *sag* mutant and the OX2 lines in medium supplemented with 200mM NaCl showed similar results to those in the mannitol-containing medium (Fig. 8D). These results suggested that AtSAG can regulate abiotic stresses during seed germination and seedling development.

## Discussion

This study demonstrated that *AtSAG*, encoding a deduced MDN1-containing protein, played an important role in ABA signalling. ABA response assays indicated that the *sag* mutant and AtSAG-RNAi (RNAi) plants were more sensitive to ABA, whereas 35S:AtSAG plants were less sensitive (Figs 1C–F, 3A–3C, and 4C), suggesting that AtSAG negatively regulated ABA signalling during seed germination and seedling development. However, downregulation of AtSAG did not obviously affect the ABA response of young seedlings (Fig. 3D), supporting the idea that AtSAG may be a ABA signalling component that was specifically effective during seed germination and early seedling development.

Studies have revealed that ABI5 is required for the germination and post-germination developmental arrest checkpoint (Lopez-Molina *et al.*, 2001). Other studies have suggested that the action of *AtSAG* in ABA signalling may be upstream of ABI5. First, the expression of *ABI5* and its target genes (*Em1*, *Em6*, *RD29A*, and *RAB18*) were upregulated in 2-d germinated seeds of the *sag* mutant, but were downregulated in OX2 (Fig. 5B–F). Second, the *sag*/*abi5* double mutant showed ABA sensitivity similar to that of the *abi5* single mutant, which was more insensitive to ABA than the *sag* single mutant and the WT seeds (Fig. 6). Finally, *AtSAG* expression was not largely changed by *abi5* mutation (Supplementary Fig. S3).

The ABI3 protein has been shown to interact with ABI5 in a yeast two-hybrid assay (Nakamura *et al.*, 2001). In this context, the current finding that *ABI3* had similar upregulated expression patterns to *ABI5* in 2-d germinated seeds of *sag* mutant but downregulated in OX2 (Fig. 5A) suggested that AtSAG may also function upstream of ABI3 in ABA signalling. Together, these data supported the idea that AtSAG was an important negative regulator of ABA signalling depending on ABI3 and ABI5 during seed germination and early seedling development.

The expressions of some seed storage protein and oleosin genes were induced by ABI3 during seed development (Crowe *et al.*, 2000; Lara *et al.*, 2003). In this study, the expression of each three selected seed storage protein and oleosin genes were induced higher by ABA in germinated seeds of *abi5* than in WT (Fig. 7), suggesting that, unlike ABI3 during seed development, ABI5 can inhibit the expression of the selected seed storage protein and oleosin genes during seed germination. These genes also showed higher induced expression levels by ABA in *sag* germinated seeds, even higher than in *abi5* (Fig. 7), suggesting that other regulators, downstream of AtSAG, functioned parallel to ABI5 or ABI3 in ABA signalling during seed germination.

Genevestigator analysis indicated that *AtSAG* was induced in the *mapk4* (mitogen-activated protein kinase 4) mutant but repressed in 35S:MKS1 (MAPK4 substrate 1) plants (Supplementary Fig. S4A). This real-time RT-PCR analysis indicated that MAPK4 showed mRNA levels upon treatment with ABA in the *sag* mutant that were not obviously different from WT (Supplementary Fig. S4B). These data suggest that AtSAG functioned parallel to or downstream of MAPK4 and MKS1. A previous study has confirmed that MAPK4 and MKS1 were associated with WRKY33 *in vivo*; necrotrophic pathogen infection led to the activation of MAPK4 and phosphorylation of MKS1, and subsequently, MKS1 and WRKY33 were released from MAPK4, and WRKY33 was recruited to the promoter of PHYTOALEXIN DEFICIENT3 (PAD3) (Qiu *et al.*, 2008). WRKY33 has been reported to be induced by 150mM NaCl treatment in *Arabidopsis* roots (Jiang and Deyholos, 2006), and *wrky33* was slightly more sensitive to NaCl treatment in a root elongation assay (Jiang and Deyholos, 2009). Therefore, AtSAG may be regulated by MAPK4, MKS1 and WRKY33 by similar pattern of PAD3.

*AtSAG* encoded a deduced MDN1-containing protein containing one VWA domain located after the only AAA domain at the N-terminus and a divergent C-terminus (Fig. 1A and Supplementary Fig. S1). The proteins containing AAA and VWA domains often functioned in the assembly of multiprotein complexes. Overexpression of MdMAPK1 from apple led to ABA sensitivity by ABI5 phosphorylation during transgenic *Arabidopsis* seed germination (Wang *et al.*, 2010). Thus, AtSAG may be involved in the assembly of protein kinase and ABI5 complexes to activate downstream gene expression. By contrast, oleosin deficiency retarded germination in *Arabidopsis* (Siloto *et al.*, 2006; Shimada and Hara-Nishimura, 2010). Major depletion of seed storage proteins resulted in the completion of germination (Angelovici *et al.*, 2011). Thus, AtSAG may be involved in post-transcriptional other than transcriptional regulation of these genes. Further analysis of one obviously different protein band that was coimmunoprecipitated by AtSAG:GFP (Supplementary Fig. S5) revealed that proteins encoded by *Olesin2* (*Ole2*), At3g01570, and At4g36700 can be coimmunoprecipitated with AtSAG:GFP with molecular weight about 30kDa. However, the predicted molecular weight of these proteins is 21, 19, and 59kDa respectively. This result confirmed the post-transcriptional regulation of AtSAG to these genes.

Two regulatory patterns for AtSAG are possible. First, the yeast MDN1-containing protein Rea1 had an important function in ribosome maturation (Bassler *et al.*, 2010). ScMDN1 and AtMDN1, the plant homologues of Rea1, were predicted to function in similar pattern to Rea1 (Chantha and Matton, 2007). If AtSAG functioned in the similar pattern, it may regulate seed storage protein and oleosin genes at the translational level. Second, RPN10 was one of the subunits of the regulatory particle (RP) of 26S proteasome that contained a VWA domain (Voges *et al.*, 1999) and was originally identified by its ability to bind polyubiquitin chains *in vitro* (van Nocker *et al.*, 1996). The *Arabidopsis rpn10-1* mutant exhibited highly sensitivity to ABA, salt, and sucrose stress by failure to specifically and rapidly degrade the ABA response protein ABI5 during early seedling development (Smalle *et al.*, 2003). The larger molecular weight of the two proteins that were coimmunoparticipated by AtSAG:GFP might resulted from the binding of polyubiquitin chains. Therefore, AtSAG may alternatively function in the assembly of 26S proteasome to degrade seed storage proteins and oleosins. However, the exact molecular process of AtSAG in ABA signalling must be investigated by discovering the interacting proteins.

## Supplementary material

Supplementary data are available at *JXB* online.

Supplementary Table S1. Primers used in this study.

Supplementary Fig. S1. BlastP alignment between AtSAG and other homologous proteins from *Glycine max*, *Vitis vinifera*, and *Ricinus communis*.

Supplementary Fig. S2. Accumulation of ABI3 and ABI5 in *sag* mutant germinated seeds with 0.5 μM ABA.

Supplementary Fig. S3. Expression analysis of *AtSAG* in *abi5* germinated seeds.

Supplementary Fig. S4. Expression analysis of *AtSAG* in *mapk4* and 35S:MKS1 plants by genevestigator analysis and expression of MAPK4.

Supplementary Fig. S5. SDS-PAGE of proteins coimmunoprecipitated by AtSAG:GFP.

Supplementary Data
