# Supplementary material for: Arabidopsis SAG protein containing the MDN1 domain participates in seed germination and seedling development by negatively regulating ABI3 and ABI5
Source: J Exp Bot. 2013 Oct 25;65(1):35–45. doi: 10.1093/jxb/ert343 (PMC3883281; doi:10.1093/jxb/ert343)
Supplement: Supplementary Data [file supp_65_1_35__index.html]

 Arabidopsis SAG protein containing the MDN1 domain participates in seed germination and seedling development by negatively regulating ABI3 and ABI5 — Arabidopsis SAG protein containing the MDN1 domain participates in seed germination and seedling development by negatively regulating ABI3 and ABI5 — Supplementary Data 

# *Arabidopsis* SAG protein containing the MDN1 domain participates in seed germination and seedling development by negatively regulating ABI3 and ABI5

## Supplementary Data

Data files

**Files in this Data Supplement:**

- Supplementary Data - Supplementary Data
