# Supplementary material for: Arabidopsis SAG protein containing the MDN1 domain participates in seed germination and seedling development by negatively regulating ABI3 and ABI5
Source: J Exp Bot. 2013 Oct 25;65(1):35–45. doi: 10.1093/jxb/ert343 (PMC3883281; doi:10.1093/jxb/ert343)

Legends for supplemental data

Table S1 The list of primers used in this study. F and R indicate forward and reverse primers.

Fig. S1 The BlastP between AtSAG and other homologous proteins from *Glycine max* (XP_003522398.1), *Vitis vinifera* (XP_002282819.2), and *Ricinus communis* (XP_002515436.1). The asterisk marked letters indicates the VWA domain components, the straight lines indicate regions of AAA and VWA domains in MDN1domain, and the black arrow indicates the T-DNA insertion site.

Fig. S2 The protein accumulation of ABI3 and ABI5 in WT and *sag* mutant seeds germinated for 2.5-day on 1/2 MS with or without 0.5 μM ABA. The black arrows in the right side indicate ABI3 and ABI5, respectively. – and + indicate without and with 0.5 μM ABA, respetively.

Fig. S3 The expression analysis of *AtSAG* in *abi5* germinated seeds compared to WT.

Fig. S4 The expression analysis of *AtSAG* in *mapk4* and 35S:MKS1 plants by genevestigator analysis (A), and the expression of MAPK4 in WT and *sag* mutant seeds germinated for 2.5-day on 1/2 MS with or without 0.5 μM ABA (B).

Fig. S5 The SDS-PAGE of coinmmunoparticipaed proteins from 35S:AtSAG-GFP and 35S:GFP seeds germinated for 2.5-day on 1/2 MS with 0.5 μM ABA**.** 1 and 2 indicated two independent repeats. The sequenced bands were marked in red box.

Supplementary data

Table S1 The list of primers used in this study.

Primer Name	Primer Sequence
NCED3-F	GCTGCGGTTTCTGGGAGAT
NCED3-R	GTCGGAGCTTTGAGAAGACGAT
NCED5-F	CCTCCGTTAGTTTCACCAACACT
NCED5-R	GGTGTGTCGGAGACGGAGTT
CYC707A1-F	CTCACTCTCTTCGCCGGAAG
CYC707A1-R	GGAGGGAGTGGGAGTTTGGAA
CYC707A2-F	CGTCTCTCACATCGAGCTCCTT
CYC707A2-R	GAGGGTGTTGATGGACTTTTGG
ABI3-F	TACTCCGTTTGTGTTCCTCAG
ABI3-R	GTTTCTCCGATTTGGGTTTGG
ABI5-F	AACCTAATCCAACCCGAACC
ABI5-R	ACCCTCCTCCTCCTGTCC
EM1-F	TCAAATGGTATGCGGTTATG
EM1-R	TATCACAAGTAAGACACGAAG
EM6-F	TGTCTCGTTTGTTTTCCAG
EM6-R	CACTATGTTGAGAATCCAC
RD29A-F	TGATCGATGCACCAGGCGTAAC
RD29A-R	CCCTGGTGGAATAATTTCCTCCG
RAB18-F	GAGCACCACGAGAAGAAGG
RAB18-R	GCACAATACAACGACCGAATG
ATS1-F	ATTACGGACTTAGTGTTC
ATS1-R	CTTGTGTATGTTGTGTATG
Ole2-F	CCACTATTTCTCCTCTTC
Ole2-R	TTCTCCTCTTAGCATACT
Ole4-F	AACTACGAAGATGATGTC
Ole4-R	GCTATAAGTGCCAAGATT
At3G22640-F	AAGGACAAGAAGAAGAAG
At3G22640-R	CGAGAGTATAGTGAATGG
At4G36700-F	GCTGTTATCTTGGAATGT
At4G36700-R	CTTCTCTTCTTCTTCTTCA
At2S4-F	CCAATACAGAAATGTCAGA
At2S4-R	GATGTCGTCTTCCATATC

Fig. S1

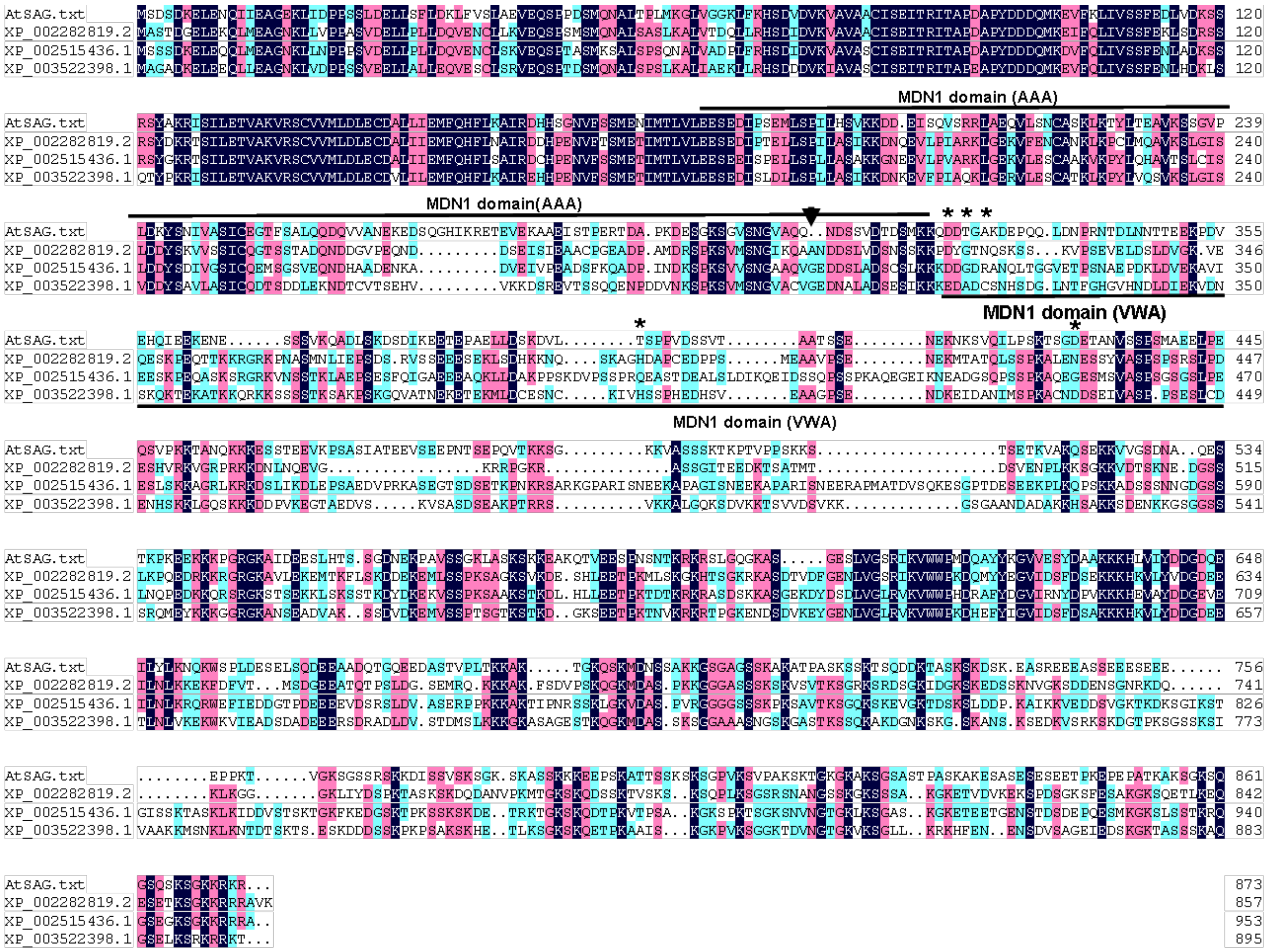
MDN1 domain (AAA)
MDN1 domain(AAA)
MDN1 domain (VWA)
MDN1 domain (VWA)
AtSAG.txt
XP_002282819.2
XP_002515436.1
XP_003522398.1

Fig. S2

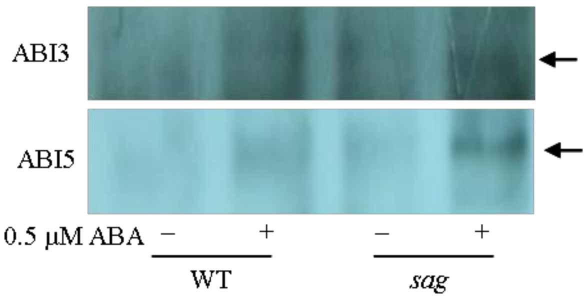

Fig. S3

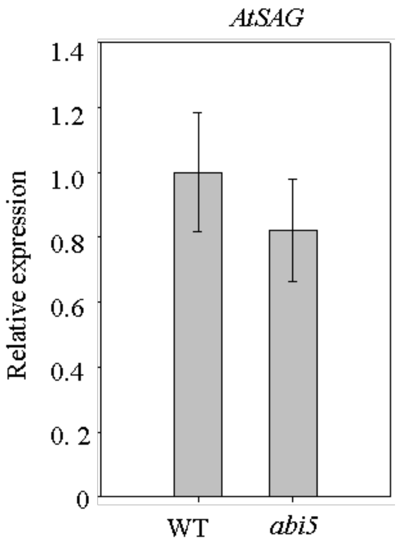

Fig. S4

A

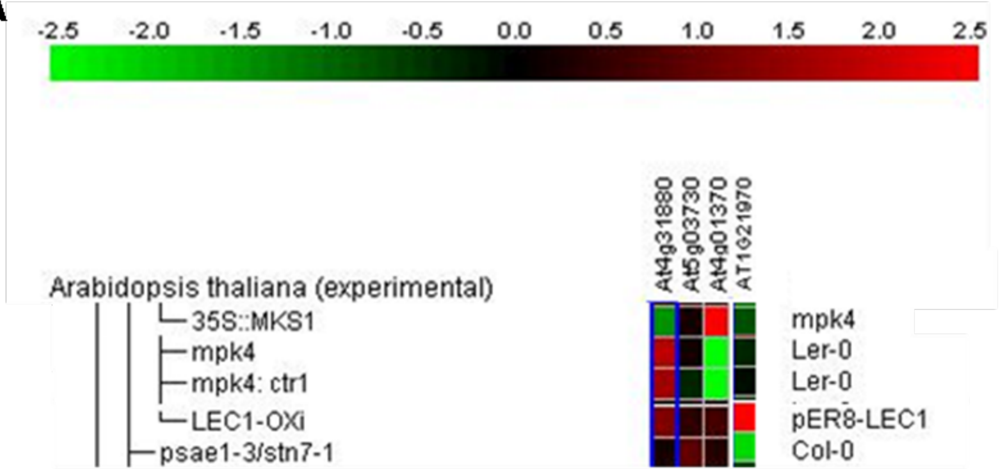
-2.5 -2.0 -1.5 -1.0 -0.5 0.0 0.5 1.0 1.5 2.0 2.5
At4g31880
At5g03730
At4g01370
AT1G21970
Arabidopsis thaliana (experimental)
35S::MKS1
mpk4
mpk4: ctr1
LEC1-OXi
psae1-3/stn7-1
mpk4
Ler-0
Ler-0
pER8-LEC1
Col-0

B

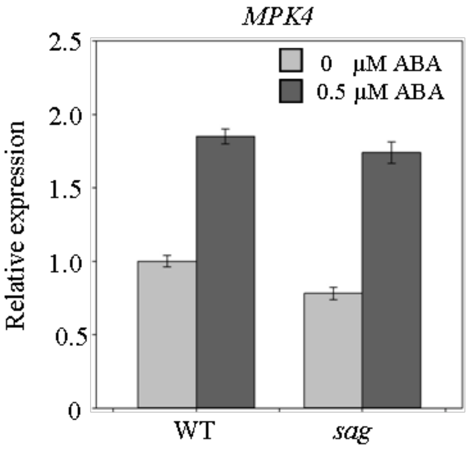
MPK4
Relative expression
2.5
2.0
1.5
1.0
0.5
0
0 μM ABA
0.5 μM ABA
WT
sag

Fig. S5

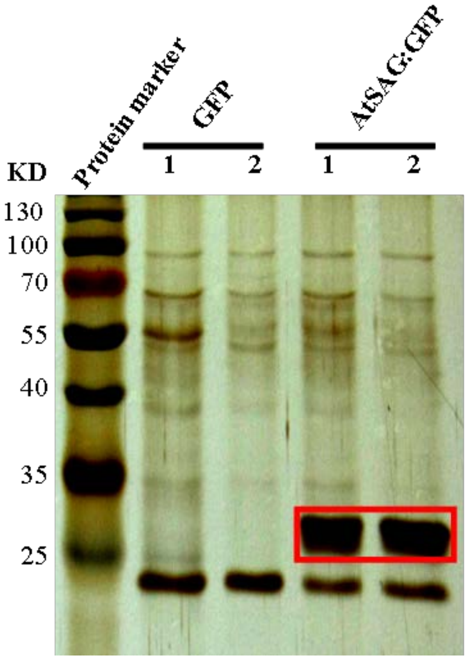

Supplement: Supplementary Data [file supp_ert343_jexbot100198_file001.pdf]
